# Assessment of Perceptions of Mental Health vs Medical Health Plan Networks Among US Adults With Private Insurance

**DOI:** 10.1001/jamanetworkopen.2021.30770

**Published:** 2021-10-22

**Authors:** Susan H. Busch, Kelly Kyanko

**Affiliations:** 1 Department of Health Policy and Management, Yale School of Public Health, New Haven, Connecticut; 2 Department of Population Health, New York University School of Medicine, New York

## Abstract

**Importance:**

Ten years after the Mental Health Parity and Addiction Equity Act, patients continue to report insurance-related barriers to specialty mental health care.

**Objectives:**

To assess privately insured patients’ perceptions of the adequacy of their health plan’s provider network (provider network includes physicians, clinicians, other health care professionals, and their institutions that constitute the network), whether practitioners frequently leave plans, and whether practitioner plan participation affected patients’ plan choice.

**Design, Setting, and Participants:**

A nationally representative, population-based internet survey study of English-speaking US adults participating in KnowledgePanel, an online research panel, was conducted from August to September 2018. Data analysis was performed from November 12, 2020, to May 12, 2021. From a sample of 29 854 panelists aged 18 to 64 years, 19 602 initiated the screener (completion rate of 66%), and 728 met study criteria: adults with private insurance receiving both specialty mental health and medical care in the past year.

**Exposure:**

Health plan’s provider network.

**Main Outcomes and Measures:**

Self-report of plan inadequacy, whether a practitioner left the plan and the participant’s responses (stopped treatment, switched practitioner, or continued treatment), and whether participation of a specific practitioner was considered when a health plan was chosen. Experiences with both mental health and medical provider networks were assessed. Analyses were weighted to match the sample to the US population. Weights provided by KnowledgePanel were also adjusted for panel recruitment, attrition, oversampling, and survey nonresponse.

**Results:**

Of a total of 728 study participants, 204 (39%) were aged 18 to 34 years, 504 (61%) were women, 82 (17%) were Hispanic, and 551 (66%) were non-Hispanic White individuals. Serious psychological distress was reported by 262 participants (36%), and 214 participants (29%) also received mental health treatment from a primary care practitioner. Participants rated their mental health provider network as inadequate more frequently than their medical provider network (163 [21%] vs 70 [10%]; odds ratio [OR], 2.69; 95% CI, 1.64-4.40; *P* < .001). However, among the 193 participants also receiving mental health treatment from a primary care practitioner, there was no significant difference in the ratings of mental health and medical provider networks (44 [14%] vs 18 [9%]; OR, 1.55; 95% CI, 0.65-3.67; *P* = .32). Sixty participants (8%) reported that a mental health practitioner had left their plan’s insurance network in the past 3 years. Of the 523 participants with a choice of plan, 98 (20%) considered whether a specific mental health practitioner was in network before choosing a plan.

**Conclusions and Relevance:**

This study’s findings suggest that more participants perceived their mental health networks to be inadequate compared with their medical networks. Increasing the availability of mental health treatment in primary care practices may aid plans in constructing adequate mental health provider networks and improve patient access to mental health care.

## Key Points

**Question:**

How do patient perceptions of mental health networks differ from perceptions of medical networks?

**Findings:**

In this survey study of 728 US adults with private insurance receiving both specialty mental health and medical care, participants were significantly more likely to rate their mental health network as inadequate compared with their medical network. Among participants also receiving mental health treatment from a primary care practitioner, there were no significant differences in perceived network adequacy.

**Meaning:**

This study’s findings suggest that increasing the availability of mental health treatment provided by primary care practitioners may aid plans in constructing adequate mental health networks.

## Teaser

This survey study assesses how patients with private insurance perceive the adequacy of their health plan’s mental health and medical provider networks, whether practitioners frequently leave plans, and whether patients’ choice of plan depends on participation by a specific practitioner.

## Introduction

Specialty mental health practitioners are more likely to opt out of participation in private insurance provider networks (provider networks include physicians, clinicians, other health care professionals, and their institutions that constitute the network), compared with practitioners in other specialties because of a combination of workforce shortages, low reimbursement compared with other specialties, and high demand for services.^1,2,3^ One study estimated that 35% of psychiatrists do not participate in managed care networks compared with 8% to 12% of other specialists.^4^ This has resulted in substantially higher rates for out-of-network mental health care compared with general medical or surgical care not related to mental health (hereinafter referred to as *medical care*), as well as concomitant higher out-of-pocket health care spending.^1,5,6^ Implications for access to care for individuals with mental health disorders may be substantial.

Network adequacy has been recognized as a necessary component of a high-quality plan.^7,8^ If there are too few practitioners in the network, enrollees may have difficulty locating an in-network practitioner who is accepting new patients within an acceptable time frame. Most states (29) have adopted at least 1 required measure of network adequacy for private health insurance plans, such as geographic distance to practitioners, time to appointment for new patients, or practitioner to enrollee ratios.^9^ However, there is a lack of consensus on how network adequacy should be measured and regulated. Even with mental health provider network requirements in place, state oversight of these regulations can be inconsistent, and compliance is often difficult to measure.^10,11,12^

Limited practitioner participation also has implications for continuity of care. Patients often have ongoing relationships with practitioners. When these practitioners no longer participate in the network either because of voluntary exit or termination by the plan, patient treatment plans may be interrupted. This may be particularly problematic for patients receiving mental health care if treatment plans are of longer duration or if practitioners need time to gain patient trust for patients to be more likely to reveal relevant sensitive information, increasing the value of continuity.^13^ Patients’ responses to a practitioner’s exit or termination from a plan may include continuing to see the same practitioner (often at higher out-of-pocket cost and less often); switching practitioners, which requires a new relationship be developed; or stopping treatment, perhaps because of frustration.

Plan choice may also be affected by limited or inadequate plan networks. Before choosing a plan, individuals may attempt to identify whether a specific practitioner is in the network. This may lead to risk selection, or a concentration of patients with the most serious conditions in a single plan. For example, plans that cover the most expensive, or star, hospitals are often chosen by the patients with the most serious illnesses or those who more frequently use these high-priced hospitals.^14^ Thus, including these hospitals can lead the cost of premiums to spiral upward, with only individuals who have the highest health care costs ultimately choosing the plan. In the case of mental health, insurance companies may attempt to avoid coverage of certain patients who require high-cost treatment by skimping on certain services or distorting network choices to avoid inclusion of certain patients (ie, excluding practitioners who provide high-cost care).^15,16,17^ Both of these concerns are particularly worrisome if patients actively consider the inclusion of specific practitioners when they choose plans.

Delivery systems have responded to mental health workforce shortages through adoption of team-based models that effectively treat conditions such as anxiety and depression through primary care practices,^18^ although in real-world settings, implementation of and fidelity to these models may be challenging because of lack of financial resources, technical guidance, and staff.^19,20,21^ In practice, primary care practitioners may address deficiencies in provider networks in several ways: they may prescribe medication for patients with mild to moderate symptoms while working collaboratively with a therapist who provides counseling, they serve as a trusted referral source and provide initial treatment while helping patients locate a specialist, or they may prescribe medication out of necessity if a patient is unable to find a specialist. Few studies have assessed whether treatment of mental health disorders by primary care practitioners has helped address mental health workforce supply issues, particularly from the perspective of patient experiences with plan provider networks.

Our objective was to compare patient perceptions and experiences with mental health and medical provider networks. We conducted a survey study of patients in the US who were receiving both types of care in the past year. We assessed patient ratings of the inadequacy of the provider network, whether in the past 3 years a treating practitioner left their network and the resulting responses, and whether practitioner participation in the network affected plan choice.

## Methods

### Survey Development

Data were obtained from a de novo 2018 national internet survey of adults with private insurance on their experiences with access to outpatient mental health services, conducted from August to September 2018. Data analysis was performed from November 12, 2020, to May 12, 2021. Survey methods have been described previously.^22^ We developed the survey, which was then tested using 10 cognitive interviews. Revisions to the survey were made when there were concerns about interpretation or survey wording.^23^ The survey was piloted online with a small number of participants. The Yale Human Investigation Committee and the New York University School of Medicine Institutional Review Board approved the study, and participants viewed a statement of consent before initiating the survey. This study followed the American Association for Public Opinion Research (AAPOR) reporting guideline.

A series of screener questions was used to identify this study sample of US adults aged 18 to 64 years who were enrolled in private insurance with a provider network and who were treated by both an outpatient mental health practitioner and an outpatient medical practitioner in the preceding 12 months. We selected participants receiving treatment from both practitioner types for these analyses to allow for within-person comparisons in patient experiences with their mental health and medical provider networks. Mental health practitioners were defined as “professionals specifically trained to diagnose and treat emotional or mental health problems, including psychiatrists, therapists, psychologists, mental health nurse practitioners, and social workers.” Medical practitioners were defined as “doctors, nurse practitioners, and physician assistants.”

We explored patient experiences with network composition through a series of questions on network adequacy, network continuity, and plan choice. When assessing network adequacy, we also considered whether results differed for individuals receiving mental health care from a primary care practitioner in addition to a specialty practitioner, those with and without serious psychological distress, those with and without self-reported fair or poor health status, and those receiving and not receiving any out-of-network mental health treatment in the past year. Serious psychological distress was assessed as a score of 13 or higher on the Kessler 6-Item Psychological Distress Scale.^24^

First, we assessed perception of mental health network adequacy by asking participants to rate, on a 5-point Likert scale—from strongly agree to strongly disagree—the statement, “My insurer has done a good job of making enough in-network mental health providers available.” We specifically noted to only include experiences with mental health practitioners or networks. We asked the same question, with medical substituted for mental health. We coded ratings of inadequate or insufficient networks by creating a binary variable equal to 1 if the participant chose disagree or strongly disagree.

Next, to assess network continuity, we asked whether the participant had a practitioner leave their network in the last 3 years and, if so, how it affected treatment (eg, the participant stopped treatment, switched practitioners, or continued to see the same practitioner). We did not distinguish whether practitioners were terminated from a network or exited voluntarily. We asked this question for 3 practitioner types (ie, mental health, specialist, and primary care), and participants could answer yes for more than 1 practitioner type. Specialists were defined as “a provider who specializes in a particular medical field. For example, dermatologists specialize in skin disorders; cardiologists specialize in problems of the heart.”

Finally, we assessed whether networks affected plan choice by asking participants if they looked up a practitioner or provider before choosing a health insurance plan, and, if the answer was yes, the practitioner or provider type (ie, mental health, specialist, or primary care practitioner or hospital). Participants could choose more than 1 practitioner or provider type. Those who looked up a practitioner or provider were then asked whether it affected their choice of plan.

Demographic information had previously been collected by KnowledgePanel. This included Health Insurance Marketplace plan participation and self-reported health status. Participants selected their race and ethnicity from a list of 5 options defined by KnowledgePanel: Black Non-Hispanic; Hispanic; White Non-Hispanic; Other, Non-Hispanic; or 2 or more races Non-Hispanic. Because of the small sample sizes we combined the Black Non-Hispanic, Other Non-Hispanic, and 2 or more races Non-Hispanic categories. Questions used for these analyses are in the eMethods in the Supplement.

### Survey Administration

We recruited participants from KnowledgePanel, a validated panel of approximately 50 000 households constructed through high-quality probability-based sampling (eFigure in the Supplement).^25^ Among the 29 854 sampled panelists, 19 602 (excluding breakoffs) completed the screening questionnaire, resulting in a survey completion rate of 66% based on the AAPOR standard definition for probability-based internet panels.^26^

All reported analyses were weighted to match participants to the US population based on current population survey data in terms of sex, age, race and ethnicity, educational level, census region, household income, home ownership, and metropolitan area. Weights provided by KnowledgePanel were also adjusted for panel recruitment, attrition, oversampling, and survey nonresponse.

### Statistical Analysis

Conditional (fixed-effects) logistic models with statistical significance set to *P* < .05 (2-sided) were used to examine relevant within-person associations. All analyses used Stata, version 16.1 (StataCorp LLC).

## Results

Of a total of 728 study participants, 204 (39%) were aged 18 to 34 years, 504 (61%) were women, 224 (39%) were men, 82 (17%) were Hispanic, 95 (17%) were non-Hispanic non-White, and 551 (66%) were non-Hispanic White. Serious psychological distress was reported by 262 participants (36%), and 214 participants (29%) also received mental health treatment from a primary care practitioner. Of these participants, 201 (21%) had household incomes of less than $50 000; and 53 (6%) were enrolled in a Marketplace plan (Table 1). Treatment by an out-of-network mental health practitioner was more common than treatment by an out-of-network medical practitioner; 354 participants (33%) received out-of-network mental health treatment, and 153 (19%) received out-of-network medical treatment (odds ratio [OR], 3.59; 95% CI, 2.25-5.73; *P* < .001). Respondents differed from nonrespondents and were more likely to be non-Hispanic White individuals (14 422 [74%] vs 6201 [60%]) and in our middle age category, aged 50 to 64 years (10 172 [52%] vs 3774 [37%]), with higher household income and levels of education, although after weighting, characteristics were more similar to the US population, as previously reported.^22^

**Table 1.** Characteristics of the Study Sample

Characteristic	No. (weighted %) (n = 728)^a^
Demographic characteristics	
Age group, y	
18-34	204 (39)
35-49	244 (35)
50-65	280 (26)
Female	504 (61)
Male	224 (39)
Race and ethnicity^b^	
Hispanic	82 (17)
Non-Hispanic	
Non-White	95 (17)
White	551 (66)
Annual household income, $	
<50 000	201 (21)
≥50 000	527 (79)
Enrolled in Marketplace plan^c^	53 (6)
Nonmetropolitan statistical area status	61 (9)
Region	
Northeast	142 (20)
Midwest	182 (22)
South	236 (33)
West	168 (25)
Health status	
Serious psychological distress^d^	262 (36)
Self-reported fair or poor health status^c^	95 (12)
Health care service use	
Received mental health care from primary care practitioner^c^	214 (29)
Treated by an out-of-network mental health specialty practitioner in the past year^e^	354 (33)
Treated by an out-of-network medical practitioner in the past year	153 (19)

^a^ Sample included participants reporting at least 1 specialty mental health practitioner visit and at least 1 medical practitioner visit in the past year. Numbers represent unweighted survey participants. Percentages were weighted to account for oversampling, survey recruitment, and nonresponse and to match the sample to the US population.

^b^ KnowledgePanel provided 5 categories for self-report: Black Non-Hispanic, Hispanic, White Non-Hispanic, Other Non-Hispanic, or 2 or more races Non-Hispanic. Because of small sample sizes we combined Black Non-Hispanic, Other Non-Hispanic, and 2 or more races Non-Hispanic categories.

^c^ Participants were omitted if any relevant questions had missing data. Marketplace plan: n = 697; self-reported health status: n = 713; mental health treatment from primary care practitioner: n = 719; all other variables n = 728.

^d^ Serious psychological distress defined as Kessler 6-Item Psychological Distress Scale score of 13 or greater.^24^

^e^ Conditional (fixed-effects) logistic regression indicates treatment by an out-of-network mental health practitioner was more common than treatment by an out-of-network medical practitioner (OR, 3.59; 95% CI, 2.25-5.73; *P* < .001).

### Network Adequacy

**Figure 1.**
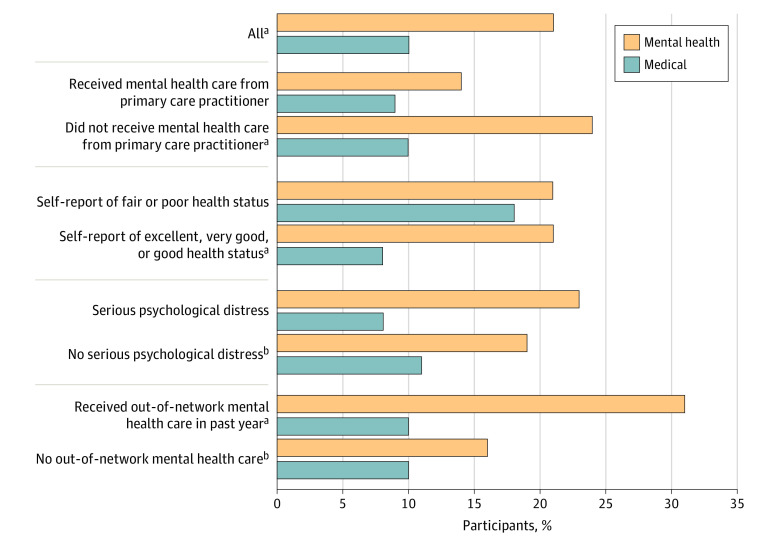
Percentage of 615 Participants Rating Their Plan’s Provider Networks as Inadequate Percentages were weighted to account for oversampling, survey recruitment, and nonresponse and to match the sample to the US population. See the Survey Development subsection of the Methods section for additional information on the screener questions and scoring. Conditional (fixed-effects) logistic regressions were used to estimate whether there was a significant difference in rating between mental health and medical provider network inadequacy within groups. Full results are shown in the eTable in the Supplement. ^a^*P* < .01. ^b^*P* < .05.

The final sample for the network adequacy analysis omitted individuals who responded “don’t know” or refused to answer, leaving 615 participants for the analysis. Overall, participants were more likely to rate their plan’s mental health provider network as not adequate compared with their plan’s medical provider network (Figure 1) (163 [21%] vs 70 [10%]; OR, 2.69; 95% CI, 1.64-4.40; *P* < .001). Although the 419 participants receiving mental health treatment only from a mental health practitioner were more likely to rate their mental health network as inadequate compared with their medical network (118 [24%] vs 52 [10%]; OR, 3.46; 95% CI, 1.91-6.28; *P* < .001), there was no significant difference in the ratings of mental health and medical provider networks among the 193 individuals also receiving mental health treatment from a primary care practitioner (44 [14%] vs 18 [9%]; OR, 1.55; 95% CI, 0.65-3.67; *P* = .32). Even among the 338 individuals only receiving in-network mental health care in the past year, mental health networks were significantly more likely to be rated as inadequate (47 [16%] vs 31 [10%]; OR, 1.92; 95% CI, 1.03-3.58; *P* = .04).

### Network Discontinuity and Consequences

Of the 523 participants with a choice of plan, 168 (21%) reported that at least 1 practitioner had left their plan’s insurance network in the past 3 years (Table 2). Participants were no more likely to report that a mental health practitioner had left their network as compared with a primary care practitioner (60 [8%] vs 80 [11%]; OR, 0.65; 95% CI, 0.40- 1.05; *P* = .08) or a specialist (60 [8%] vs 57 [7%]; OR, 1.18; 95% CI, 0.68- 2.02; *P* = .56). Among those who reported that a practitioner left their network, for all practitioner types, switching practitioners was the most common response: 63 of 80 participants (69%) switched primary care practitioners, 23 of 60 (40%) switched mental health practitioners, and 27 of 55 (44%) switched specialty practitioners.

**Table 2.** Network Discontinuity and Participant Responses, by Practitioner Type

	Practitioner type		
	Mental health	Primary care	Specialty
Respondents, No.	708	708	708
Practitioner left insurance plan’s network in the past 3 y, No. (%)^a^	60 (8)	80 (11)	57 (7)
Response to practitioner leaving the network, No. (%)			
Respondents, No.	60	80	55
Switched practitioner	23 (40)	63 (69)	27 (44)
Continued to see practitioner	25 (34)	14 (24)	18 (25)
Stopped treatment	12 (26)	3 (7)	10 (31)

^a^ Percentages were weighted to account for oversampling, survey recruitment, and nonresponse and to match the sample to the US population. In total, 168 (21%) reported that at least 1 practitioner left their plan’s insurance network in the past 3 years. Conditional (fixed-effects) logistic regression tests for differences in practitioner leaving network: mental health practitioner vs primary care practitioner: odds ratio (OR), 0.65; 95% CI, 0.40-1.05; *P* = .08; mental health practitioner vs specialty practitioner: OR, 1.18; 95% CI, 0.68-2.02; *P* = .56; primary care practitioner vs specialist: OR, 1.82; 95% CI, 1.13-2.92; *P* = .01. Full results are in the eTable in the Supplement.

### Network Composition and Plan Choice

**Figure 2.**
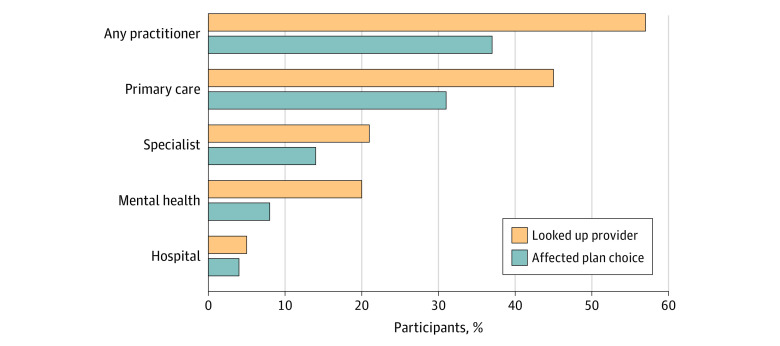
Percentage of 523 Participants Who Looked Up a Practitioner Before Choosing a Plan and Whether It Affected Plan Choice Percentages were weighted to account for oversampling, survey recruitment, and nonresponse and to match the sample to the US population. See the Survey Development subsection of the Methods section for additional information on the questions and scoring. Conditional (fixed-effects) logistic regression tests for differences across practitioner types are in the eTable in the Supplement.

Among the 523 participants who had a choice of plan in our sample, 302 (57%) considered whether at least 1 practitioner participated in the network before choosing a plan (Figure 2). Participants were most likely to check the network status of a primary care practitioner (236 [45%]). The percentages of participants who checked the status of a specific mental health practitioner and a specialty practitioner were similar (98 [20%] and 124 [21%]). In our sample, only 35 participants (5%) considered whether a specific hospital was in a network before choosing a plan.

For 200 participants (37%), the availability of a practitioner in the network influenced the choice of plan. However, among our sample of individuals who received mental health services in the past year, only 46 (8%) responded that the inclusion of a specific mental health practitioner ultimately influenced plan choice.

## Discussion

We found that patients with private insurance were more likely to rate their mental health provider network (“provider network” includes physicians, clinicians, other health care professionals and their institutions that comprise the network), as inadequate compared with their medical provider network. Although according to The Paul Wellstone and Pete Domenici Mental Health Parity and Addiction Equity Act^27^ mental health networks must use the same provider admission standards as medical networks, network adequacy can be challenging to measure and enforce. Listed practitioners may have inaccurate contact information or no longer participate in the plan, may not be accepting new patients, or may have long wait times for appointments, resulting in so-called ghost networks.

Although the high rates of treatment by out-of-network mental health practitioners and patient perception of disparities in network adequacy between mental health and medical plan networks that we found in this study are not de facto evidence of inadequate mental health networks, they may be a signal of inadequate networks. States often rely on information from consumers to identify network adequacy issues, although few enrollees submit formal complaints.^22^ Stronger quantifiable network adequacy standards that include wait times, whether practitioners are accepting new patients, and whether practitioners have submitted a behavioral health claim in the past 6 months may improve access.^28^

We did not find differences in the ratings of mental health and medical network adequacy among those receiving treatment from primary care practitioners, and, to our knowledge, this finding has not been reported earlier. Increased resources devoted to primary care practitioners may help insurers address deficiencies in mental health networks for plan enrollees, including payment mechanisms that adequately reimburse primary care physicians for providing mental health care, increased technical guidance for practitioners, and additional resources for staff to provide care management services. This finding suggests that when mental health network adequacy is being assessed, the availability of primary care practitioners to fill this role, such as those participating in collaborative care models, might be considered.^28^

We found that patients were no more likely to experience the exit of a mental health practitioner than the exit of a primary care or specialty practitioner. Regulations related to consumer protections when practitioners are terminated vary considerably by state and do not apply to self-funded plans.^29^ Although the final regulations have not been released to date, the No Surprises Act (2020) addresses this issue in group health plans in part, allowing some patients in active treatment to continue under in-network cost sharing for 90 days when a practitioner is terminated without cause.^30^ Whether all mental health care will be covered under this provision has not yet been determined. Other policy solutions are needed to retain (and recruit) practitioners in networks, possibly through reimbursement and a reduction in administrative burden.^31^ The high rate of denials of claims for mental health treatment under private insurance suggests this may be a relevant factor underlying low participation among mental health practitioners.^32,33^

Overall, our results were consistent with those of a survey among individuals enrolled in a single health plan,^34^ which showed that approximately 60% of participants considered inclusion of a specific doctor or hospital when choosing a plan, although the researchers did not examine whether participants considered network status of mental health practitioners. We found that individuals were no more likely to check the network status of mental health practitioners than other specialists. Checking the status of either type of practitioner was relatively uncommon; only about 20% of individuals did so, and even fewer noted that this information affected their plan choice. This suggests that concerns about plan selection through network construction may be less common than originally believed. Researchers have previously found that enrollees are willing to pay more than $1000 annually for a plan that includes a current practitioner, although those researchers also found evidence of high rates of “inattention bias,” with many enrollees failing to choose a lower-cost plan that includes their current practitioner.^35^

### Strengths and Limitations

Our study has several strengths. Network adequacy has been defined differently by states, the federal government, and plan type. We sidestepped this issue by asking patients directly whether they believe their plan’s provider network is adequate. This consumer-centric focus allowed us to directly compare at least 1 measure of network adequacy across mental health and medical networks. Comparing responses for the same participants in our sample allowed us to address concerns that individuals receiving different types of care may differ in person-level characteristics that also affect responses to care experience questions. Another strength is that we examined a national sample, and most survey participants were enrollees in non-Marketplace commercial plans, the focus of current policy.^30^

This study has limitations related to issues common to all surveys, including potential biases related to self-report and response bias. To address these issues, we used cognitive interviewing to pretest the survey instrument, and responses were weighted by demographic characteristics, yet some biases may remain. In some cases, our sample size was limited, making it more likely that we failed to detect meaningful differences between groups. If a participant received treatment more often from one practitioner type than another (eg, more treatment from specialists than from primary care practitioners), the patient might have been more likely to have a negative experience related to a provider network. When we considered whether the provider network affected plan choice, we were unable to identify the patients whose treatment had the highest cost.

It is notable that our study sample does not consider the experiences of patients who attempted but did not ultimately receive treatment, arguably the group that had the most difficulty locating a practitioner. The survey included only individuals with private insurance; thus, perceptions of network adequacy for Medicaid managed care plans or Medicare Advantage plans were not assessed.

## Conclusions

In this survey study, more respondents perceived their mental health provider networks to be inadequate than those who perceived their medical provider networks to be inadequate. This finding suggests a need for better enforcement of existing laws or new regulations to ensure more consistent access to affordable mental health treatment. In addition, increasing availability of evidence-based mental health treatment in primary care may aid plans in constructing adequate mental health provider networks.

## Notes

Supplement. **eMethods.** Survey Questions **eFigure.** Sample Selection **eTable.** Statistical Test Results
