# Incorrect Grant Number in Funding/Support

**DOI:** 10.1001/jamanetworkopen.2022.27336

**Published:** 2022-07-26

In the Original Investigation titled “Assessment of Perceptions of Mental Health vs Medical Health Plan Networks Among US Adults With Private Insurance,”^1^ published October 22, 2021, an incorrect grant number appeared in the Funding/Support entry of the Article Information section. The grant number should have been given as R21MH109783. This article has been corrected.^1^
