# Supplementary material for: Assessment of Perceptions of Mental Health vs Medical Health Plan Networks Among US Adults With Private Insurance
Source: JAMA Netw Open. 2021 Oct 22;4(10):e2130770. doi: 10.1001/jamanetworkopen.2021.30770 (PMC8536951; doi:10.1001/jamanetworkopen.2021.30770)
Supplement: Supplement. — eMethods. Survey Questions eFigure. Sample Selection eTable. Statistical Test Results [file jamanetwopen-e2130770-s001.pdf]

Supplementary Online Content

Busch SH, Kyanko K. Assessment of perceptions of mental health vs medical health plan networks among US adults with private insurance. *JAMA Netw Open*. 2021;4(10):e2130770.
doi:10.1001/jamanetworkopen.2021.30770

eMethods. Survey Questions

eFigure. Sample Selection

eTable. Statistical Test Results

This supplementary material has been provided by the authors to give readers additional information about their work.

© 2021 Busch SH et al. *JAMA Network Open*.

eMethods. Survey questions

Data were obtained from a 2018 survey on patient experiences with access to outpatient mental health services. Screener and survey questions for relevant outcome variables are presented below. For words in green, the participant was able to mouse or hover over the word for a definition, available at the end of this Appendix.

Screener

For more detail on sample selection see eAppendix 2. A series of screener questions was sent to panelists ages 18-64 to identify participants enrolled in a private health insurance plan with a provider network. Our study sample consisted of privately insured English-speaking adults ages 18-64 reporting that they used both an outpatient mental health provider in the last year and an outpatient medical provider in the last year.

The following questions were used to obtain the sample. The first three questions restricted the sample to individuals with private health insurance with a provider network that had seen a mental health provider in the past year. We oversampled individuals that had used out-of-network mental health care (determined by the fourth question noted).

1. These questions ask about **your own** health care, not a family member's. If you need help understanding one of the words that is in green, you can hover over it for more information.

   Private health insurance can be through an employer or union, bought directly by you, or bought from a state or federal health insurance marketplace. It does *not* include private Medicare or Medicaid plans.

   Do you currently have **private health insurance?**
   - o Yes
   - o No

2. An insurer **provider network** is a group of doctors or hospitals that takes your insurance. Usually you pay less (or nothing) for services from providers in the network.

   Does your health insurance plan have a **provider network**?
   - o Yes
   - o No

3. A **MENTAL HEALTH provider** is a professional specifically trained to diagnose and treat emotional or mental health problems, including psychiatrists, therapists, psychologists, mental health nurse practitioners, and social workers.

   In the last 12 months, have you seen a **MENTAL HEALTH** provider **in an office or clinic or by telemedicine**?
   - o Yes
   - o No

4. A provider is considered **out-of-network** if they are not in your insurer's **provider network**. Some of these providers do not accept any private insurance.

   Were any of these **MENTAL HEALTH** providers you saw **out-of-network**?
   - o Yes
   - o No

5. A **MEDICAL provider** is a doctor, nurse practitioner, or physician assistant. For this survey, it does not include mental health providers, including psychiatrists. For this survey, it also does not include dental, chiropractic, naturopathic, or homeopathic care.

   In the last 12 months, have you seen a **MEDICAL** provider **in an office or clinic or by telemedicine**? Do not include providers you saw in the emergency room or while overnight in the hospital.
   - o Yes

© 2021 Busch SH et al. *JAMA Network Open*.

- No

6. Were any of these **MEDICAL** providers you saw **out-of-network**?
   - Yes
   - No

Questions related to network adequacy

Do you agree or disagree with the following statements?

My insurer has done a good job of making enough in-network **mental health providers** available.

- Strongly disagree
- Disagree
- Neither agree or disagree
- Agree
- Strongly agree
- Don't know

Do you agree or disagree with this statement?

My insurer has done a good job of making enough in-network **medical providers** available. Please do not consider your experiences with your insurer's mental health provider network.

- Strongly disagree
- Disagree
- Neither agree or disagree
- Agree
- Strongly agree
- Don't know

We coded reports of inadequate or insufficient networks as a binary variable equal to one if the respondent chose 'disagree' or 'strongly disagree'. Participants noting "Don't know" or refused the question were coded as missing.

Questions related to plan choice

Now we are going to ask you some questions about your experiences with your health insurance plan.

When choosing your insurance plan, did you try to determine if a specific provider was **in-network** before making a decision? The provider may have been for care for you or a family member. This could have been a primary care provider, mental health provider, specialist or hospital.

- Yes
- No
- Not applicable. I did not have a choice of plan.

What type of provider was it?

- Primary care provider (family doctor, general practitioner, pediatrician)
- Mental health provider
- Specialist (for example, a cardiologist or gynecologist)
- Hospital

We then asked the following question separately substituting provider type (primary care provider, mental health provider, specialist and hospital) for each provider selected in the question above.

Did the availability of the [provider type] impact your choice of insurance plan?

- Yes
- No

© 2021 Busch SH et al. *JAMA Network Open*.

Questions related to plan continuity

In the past three years, did you ever have a provider (either medical or mental health) you were seeing leave your insurer's provider network?
- o Yes
- o No

What type of provider was it? Check all categories that apply.
- o Mental health provider (for example, psychiatrist, psychologist, therapist)
- o Primary care provider (for example, family doctor, general practitioner, pediatrician)
- o Specialist (for example, cardiologist or gynecologist)

We then asked the following question for each provider type selected in the question above:

When the [provider type] left the insurance network, what happened? If more than one [provider type] left your insurance network, choose the one who left most recently.
- o I stopped treatment
- o I switched to different provider
- o I stayed with the same provider and saw them just as frequently
- o I stayed with the same provider but saw them less often
- o I stayed with the same provider but my insurer continued to treat as in-network

Hover over definitions for survey words in green

In-network provider*:* An in-network provider is in your insurer provider network and takes your insurance. Usually you pay less (or nothing) for services from doctors in the network.
Medical provider: A doctor, nurse practitioner, or physician assistant. For this survey, it does not include mental health providers, including psychiatrists. For this survey, it also does not include dental, chiropractic, naturopathic, or homeopathic care.
Mental health provider: A professional specifically trained to diagnose and treat emotional or mental health problems, including psychiatrists, therapists, psychologists, mental health nurse practitioners, and social workers.
Out-of-network provider: A provider not in your insurer's provider network. Some of these providers do not accept any private insurance. Usually you pay more for out-of-network providers.
Primary care provider: A primary care doctor or provider is the person or place an individual would go to if they had a new health problem, needed preventive health care, or needed a referral to a specialist. Examples of primary care providers include general practitioners, family practitioners, and internists.
Provider network: A group of doctors or hospitals that takes your insurance. Usually you pay less (or nothing) for services from providers in the network.
Specialist: A provider who specializes in a particular medical field. For example, dermatologists specialize in skin disorders, cardiologists specialize in problems of the heart, etc.
Telemedicine: Also called "telehealth". Rather than seeing a patient in person, a health provider uses remote health care technology (computer, skype) to deliver care.

© 2021 Busch SH et al. *JAMA Network Open*.

eFigure. Sample selection

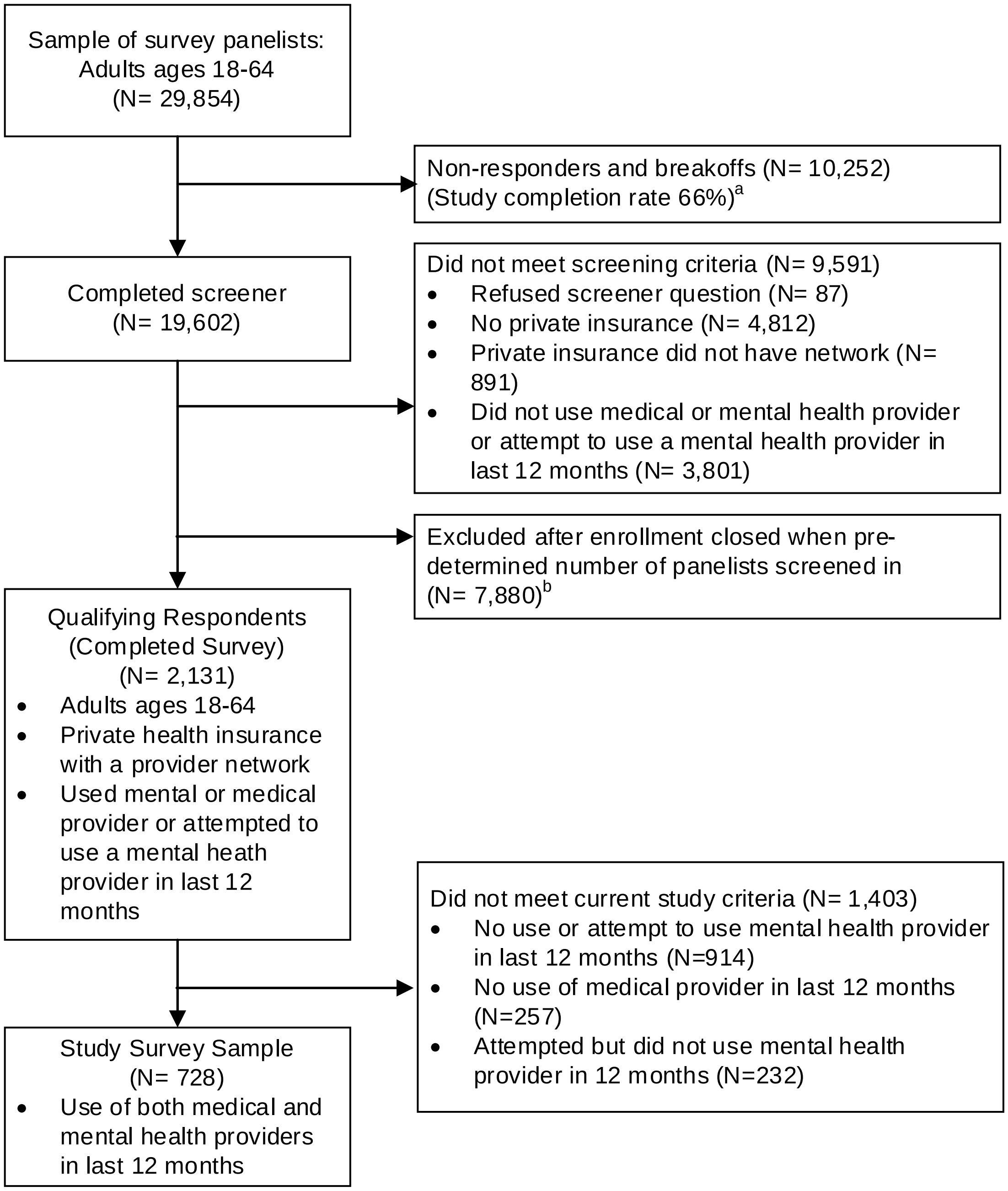

[a] Completion rate, the proportion of panelists assigned to the survey that responded to the invitation and completed the screener (excluding breakoffs), was calculated using the standard American Association for Public Opinion Research (AAPOR) definition for probability-based internet panels. Available from: https://www.aapor.org/AAPOR_Main/media/publications/Standard-Definitions20169theditionfinal.pdf.
[b] Participants who used mental health providers and out-of-network providers were oversampled. Once the predetermined quotas for general medical care and in-network care were filled, participants were no longer asked to complete the full survey.

© 2021 Busch SH et al. *JAMA Network Open*.

eTable: Statistical test results

eTable, A: Statistical test results for Figure 1. Percentage of 615 participants rating their plan's provider networks as inadequate

			Mental health versus medical provider network			Difference in difference of mental health versus medical provider network across group		
		N (weighted percent)	Odds ratio	CI	p-value	Odds ratio	CI	p-value
Full Sample***	Mental Health	163 (21)						
	Medical	70 (10)	2.685	1.638- 4.400	<.001			
Received mental health care from primary care provider	Mental Health	44/193 (14)						
	Medical	18/193 (9)	1.548	.653-3.672	0.322			
Did not receive mental health care from primary care provider***	Mental Health	118/419 (24)						
	Medical	52/419 (10)	3.461	1.908-6.278	<.001	0.447	.158-1.270	0.131
Self report of fair or poor health status	Mental Health	27/78 (21)						
	Medical	19/78 (18)	1.273	.374- 4.331	0.699			
Self report of excellent, very good, or good health status***	Mental Health	135/529(21)						
	Medical	50/529 (8)	3.278	1.893-5.675	<.001	0.388	.104- 1.449	0.159
Serious Psychological Distress***	Mental Health	65/228 (23)						
	Medical	29/228 (8)	4.149	1.950- 8.827	<.001			
No Serious Psychological Distress**	Mental Health	98/387 (19)						
	Medical	41/387 (11)	2.122	1.132-3.978	0.019	1.955	.735- 5.202	0.179
Received out-of-network mental health care in past year***	Mental Health	116/277(31)						
	Medical	39/277(10)	5.231	2.107-12.991	<.001			
No out-of-network mental health care**	Mental Health	47/338(16)						
	Medical	31/338(10)	1.923	1.033-3.579	0.039	2.721	.907- 8.159	0.074

© 2021 Busch SH et al. *JAMA Network Open*.

eTable, B: Statistical test results for Table 2. Network discontinuity and participant responses, by practitioner type

	**Practitioner left insurance plan's network in the past 3 y (N=708)**		
	N (weighted %)		
Any provider	168 (21%)		
Mental health provider	60 (8)		
Primary care provider	80 (11)		
Specialty provider	57 (7)		
**Tests of comparisons**	**odds ratio**	**95% CI**	**p-value**
Mental health providers versus primary care providers	0.646	.399- 1.046	p=.076
Mental health providers versus specialists	1.175	.684- 2.018	p=.559
Primary care providers versus specialists	1.817	1.130-2.923	p=.014

© 2021 Busch SH et al. *JAMA Network Open*.

eTable, C: Statistical test results for Figure 2. Percentage of 523 participants who looked up a practitioner before choosing a plan and whether it affected plan choice

	Panel 1: Looked up a provider (N=523)			Panel 2: Impacted plan choice (N=523)		
	N (weighted percent)			N (weighted percent)		
Any provider	302(57)			200 (37)		
Primary care provider	236 (45)			164 (31)		
Mental health provider	98 (20)			46 (8)		
Specialist	124 (21)			77 (14)		
Hospital	35 (5)			24 (4)		
Comparisons	odds ratio	95% CI	p-value	odds ratio	95% CI	p-value
Mental health provider versus primary care	0.168	.096-.293	p<.001	0.053	.025-.109	p<.001
Specialist versus primary care	0.198	130-.301	p<.001	0.183	.108- .310	p<.001
Hospital versus primary care	0.014	.008-.026	p<.001	0.019	.008- .042	p<.001
Specialist versus mental health	1.179	.696-1.997	p=.541	3.481	1.760- 6.882	p<.001
Hospital versus mental health	0.084	.044-.160	p<.001	0.357	.145-.879	p<.025
Hospital versus specialist	0.071	.039-.132	p<.001	0.103	.046- .228	p<.001

© 2021 Busch SH et al. *JAMA Network Open*.
